# Genotyping of *Clostridium perfringens* isolated from broiler meat in northeastern of Iran

**Published:** 2015-12-15

**Authors:** Asma Afshari, Abdollah Jamshidi, Jamshid Razmyar, Mehrnaz Rad

**Affiliations:** 1 *Graduate Student,School of Veterinary Medicine, Ferdowsi University of Mashhad, Mashhad, Iran; *; 2 *Department of Food Hygiene, School of Veterinary Medicine, Ferdowsi University of Mashhad, Mashhad, Iran; *; 3 *Department of Clinical Sciences, School of Veterinary Medicine, Ferdowsi University of Mashhad, Mashhad, Iran; *; 4 *Department of Pathobiology, School of Veterinary Medicine, Ferdowsi University of Mashhad, Mashhad, Iran.*

**Keywords:** *Clostridium perfringens*, cpe gene, Multiplex PCR

## Abstract

*Clostridium perfringens (C. perfringens)* is an important cause of bacterial food poisoning worldwide. The disease is caused by *C. perfringens* enterotoxin (CPE) encoded by *cpe* gene. The aim of this research was to identify the different types of *C. perfringens* and the presence of *cpe* gene in isolated bacteria from broilers’ meat marketed in retail meat shops of Mashhad city in Northeastern of Iran. After isolation of *C. perfringens* using conventional culture method and confirmation by specific 16S rDNA gene, a multiplex polymerase chain reaction assay with specific primers, were performed for toxin typing of isolates. *Clostridium perfringens* was isolated from 31 broilers’ meat samples (15.50%) out of 200 samples and for toxin typing the results showed 9 isolates as type A (29.03%) and 22 isolates as type C (70.96%). In this study, *cpe*-positive *C. perfringens* were detected in eight isolates of type C (25.00%). Our results indicated that *C. perfringens* type C is the most common type in broiler chicken carcasses.

## Introduction

*Clostridium perfringens* has been classified into five types (A–E) on the basis of its ability to produce more than one of the major lethal toxins α, β, ε, and ι. Enterotoxin producing *C. perfringens* (*cpe+*) type A is reported continuously as one of the most common food poisoning agents worldwide.^1^

The diarrheic and cramping symptoms of *C. perfringens* type A food poisoning result from *C. perfringens* entero-toxin (*cpe*).^2^ This toxin is both necessary and sufficient for the enteric virulence of *C. perfringens* type A food poisoning isolates.^2^ *In vivo* production of the enterotoxin is associated with sporulation in the intestine, while in vitro production of enterotoxin is obtained in appropriate culture media.^3^ Only a small fraction (less than 5.00%) of all *C. perfringens* isolates, mainly belonging to type A carrying the *cpe* gene.^4^ The *cpe* gene can have either a chromosomal or a plasmid-borne location but is nearly always present on the chromosome of food poisoning isolates.^5^ There is strong association between type A isolates carrying a chromosomal *cpe *gene and *C. perfringens* type A food poisoning is attributable (at least in part) to the exceptional heat resistance of those isolates, which should favor their survival in incompletely cooked or improperly held foods.^2^ Some type C, D, and E, isolates also carry functional *cpe* genes on large plasmids.^6^ Surveys clearly demonstrated that *C. perfringens* isolates are often present in foods, particularly raw meats and poultry.^2^^,^^7^

For toxin detection, in some laboratories, a serum neutralization test on mice or guinea pigs is employed to determine and diagnose bacterial toxin. This method is tedious, time-consuming, expensive and monovalent. Furthermore, it is improper and unethical to apply it at the expense of laboratory animals.^8^ According to Timoney *et al., *enzyme-linked immunosorbent assays (ELISA), proved to be a specific, quick and economical method that may replace the serum neutralization test.^9^ ELISA utilizes polyclonal antibodies to identify *C. perfringens* toxins.^10^ However, its disadvantage is the interaction reaction among the produced antibodies works against the toxins, which may make the identification of toxin types difficult.^11^ Biochemical tests are also incapable of distinguishing different types of *C. perfringens.*^12^ Polymerase chain reaction (PCR) is the most modern practical technology in diagnosing infectious diseases and compared with classical techniques, it is rapid (a few hours) and more reliable.^10^^,^^13^ Various PCR protocols, including multiplex PCR assays, have been established to genotype the* C. perfringens* isolates with respect to *cpa, cpb, etx, iap, cpe* and *cpb2* genes, encoding the alpha, beta, epsilon, iota, entero and beta 2 toxins, respectively.^14^^-^^21^

As molecular typing of *C. perfringens* is important for epidemiologic surveys and since there has not been enough information about *C. perfringens* in broilers’ meat in Iran, the purpose of this study was to determine the incidence and toxin typing of *C. perfringens* in broilers’ meat collected from retail meat shops in Mashhad city of Iran.

## Materials and Methods

**Sampling. **A total of two hundred samples of broiler carcasses were collected randomly from retail meat shops, using rinse technique for recovering surface bacteria as follows: The broiler carcass was placed in a sterile 1 L plastic bag, 300 mL of phosphate buffer was added. After shaking the bag for 15 sec, the rinse suspension was transferred to laboratory on ice and began bacterial analysis within 1 to 4 hr.

**Bacterial isolation. **After filtration with sterilized cheese cloth and centrifugation at 4000 rpm for 10 min of each rinsed fluid in two 50 mL falcon tubes, 10 mL of fluid tioglicolate (FTG Difco, Detroit, USA) enrichment medium was added to each pellet. One of those two tubes was heat shocked at 72 ˚C for 20 min before anaerobic incubation at 37 ˚C for 24 hr. Each FTG enrichment culture was streaked onto one plate of nutrient agar containing 10% sheep blood and 40 μg mL^-1^ neomycin and incubated for 24 hr at 37 ˚C in an anaerobic jar (Merck, Darmstadt, Germany). The plates were examined for typical colonies of *C. perfringens*. Suspected colonies were subjected to macroscopic examination (shape, size and texture of the colonies on blood agar plates).

**Preparation of cell lysates. **A single colony of each sample was suspended in 100 μL distilled water, boiled for 10 min and then centrifuged at 10,000 rpm for 10 min. The supernatants were collected carefully and used as template DNA for PCR.

**Genus-specific PCR. **The identity of the 31 recovered isolates was confirmed as* C. perfringens* based on the species specific 16S rDNA gene PCR, using specific primers, with oligonucleotide sequence.^22^

**Toxin typing. **Six pairs of primers were used to determine the presence of *cpa*, *cpb*, *iA*, *etx*, *cpe*^16^ and *cpb2 *genes,^23^ using multiplex PCR technique for all isolates (Table 1). Two strains, *C. perfringens* CIP 106157 (cpa*+*, cpe*+*) and *C. perfringens* CIP 60.61 (*cpa+*, *cpb+*, *etx+, cpb2+*) obtained from Pasteur Institute Collection (CIP; Paris, France) were used as positive controls. Amplification reactions were carried out in 50 μL volume, containing 5 μL 10x PCR buffer, 5 mM dNTPs, 25 mM MgCl_2_, 5U of *Taq* DNA poly-merase, 0.50 mM of each *cpa* oligo, 0.36 mM of each *cpb* oligo, 0.36 mM of each *cpb2 *oligo, 0.52 mM of each *iA* oligo, 0.44 mM of each *etx* oligo, 0.34 mM of each *cpe *oligo, and dH_2_O. Template DNA (10 μL) was added to the mixture. Amplification was programmed in a thermo-cycler (Model TC3000; Techne, Duxford, UK) as follows: 95 ˚C for 3 min followed by 35 cycles at 94 ˚C for 1 min, 55 ˚C for 1 min, 72 ˚C for 1 min and a final extension at 72 ˚C for 10 min.^16^ The amplification products were detected by gel electrophoresis in 1.5% agarose gel in 1x TAE buffer**, **stained with 0.5 μg mL^-1^ ethidium bromide. Amplified bands were visualized and photographed under UV transillumination.

**Table 1:** Primers for 16S rDNAgene, *cpa, cpb, etx, iA, cpe and cpb2 *toxin genes detection

**Target gene**	**Primer sequences (5`-3`)**	**Product length (bp)**	**References**	**Annealing temperatures**
**16S rDNA**	AAAGATGGCATCATCATTCAAC TACCGTCATTATCTTCCCCAAA	279	22	53 ˚C
***cpa***	GCTAATGTTACTGCCGTTGA CCTCTGATACATCGTGTAAG	324	16	55 ˚C
***cpb***	GCGAATATGCTGAATCATCTA GCAGGAACATTAGTATATCTTC	196	16	55 ˚C
***etx***	GCGGTGATATCCATCTATTC CCACTTACTTGTCCTACTAAC	655	16	55 ˚C
***iA***	ACTACTCTCAGACAAGACAG CTTTCCTTCTATTACTATACG	446	16	55 ˚C
***cpe***	GGAGATGGTTGGATATTAGG GGACCAGCAGTTGTAGATA	233	16	55 ˚C
***cpb2***	AGATTTTAAATATGATCCTAACC CAATACCCTTCACCAAATACTC	567	23	55 ˚C

## Results

From total of 200 samples,* C. perfringens* isolated from 31 samples (15.50%) of broiler chicken carcasses and confirmed by using PCR assay amplifying a specific segment of 16S rDNA gene of *C. perfringens.*

**Fig.1:**
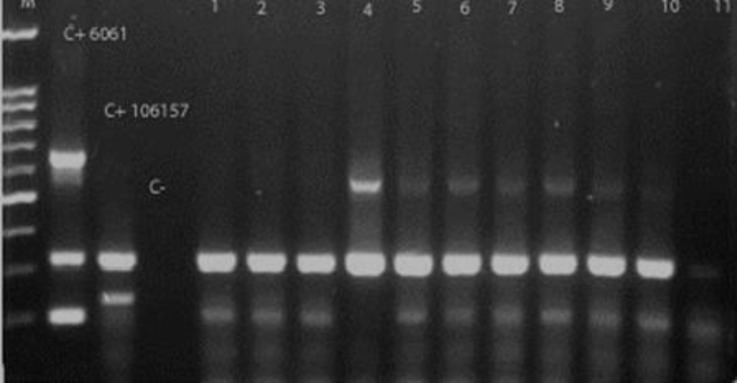
Agarose gel electrophoresis of PCR products obtained from *C. perfringens *isolated from broiler carcass samples. Lane M: Marker (DNA ladder, 100 bp); C+ CIP 60.61(*cpa+*, *cpe+*) and C+ 106157 (*cpa+*, *cpb+*, *etx+, cpb2+*): Positive controls; C-: Negative control; Lane 4: *C. perfringens *type A isolate; Lanes 1, 2, 3, 5, 6, 7, 8, 9, 10: *C. perfringens *type C isolates

**Fig. 2:**
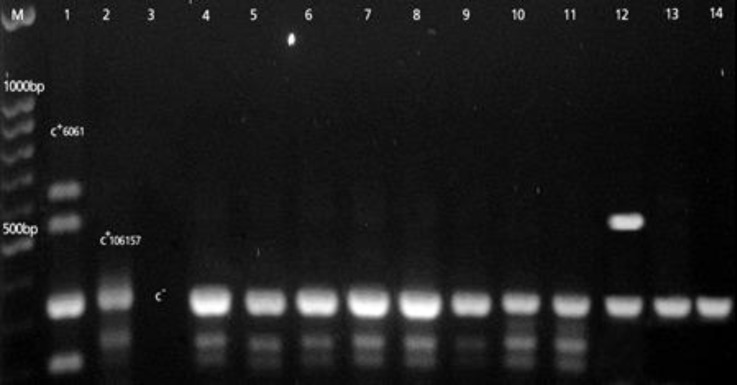
PCR identification of *cpe* gene. Lane M: Marker (DNA ladder, 100 bp); Lane 1: C+ CIP 60.61 *C. perfringens* (*cpa+*, *cpb+*, *etx+, cpb2+*) and lane 2: C+ CIP 106157: Positive controls; Lane 3 negative control; Lanes 4,5,6,7,8,9,10,11: *C. perfringens *type C isolates with *cpe* gene; Lanes 12 , 13 and 14: *C. perfringens *type A isolates

For toxin typing, the bacterial isolates were analyzed by multiplex PCR assay using specific primers in order to determine the presence of *cpa*, *cpb*, *iA*, *etx*, *cpe *and *cpb2 *genes. PCR results corresponding to positive and negative controls are displayed in Figure 2. Out of 31 *C. perfringens* isolates, 9 (29.03%) isolates were determined as type A (carrying the alpha toxin gene). From these 9 isolates, 4 (44.40%) were determined as simple type A (carrying neither the* cpe* nor *cpb2 *gene) and 5 (55.50%) isolates were determined as hetero-geneous types (carrying *cpb2 *gene) but none of the isolates were found to carry both the *cpb2 *and *cpe* genes. As the dominant type, 22 isolates (70.96%) were determined as type C (Fig. 1). 

The most important result from multiplex PCR analysis was detection of the *cpe* gene in eight isolates of *C. perfringens *which all of them belonged to type C (Fig. 2).

## Discussion

Food poisoning caused by *C. perfringens* is among the common illnesses resulting from the consumption of contaminated food and it has been firmly established that enterotoxin produced in the intestine following sporulation of ingested vegetative cells is responsible for the illness caused by *C. perfringens.*^24^

In Norway in the 1990s, *C. perfringens* was registered as the most common cause of food poisoning.^13^ The prevalence in other countries, such as Japan, the US and the UK, is also high.^13^ In England and Wales, *C. perfringens* was the second most frequently reported organism associated with food borne outbreaks of intestinal disease in the 1990s.^25^^,^^26^ The vehicles of infection are typically meat and poultry products.^2^ A survey by Lin and Labbe demonstrated these foods to be the most heavily contaminated with *C. perfringens* isolates.^27^

According to a report published in 2013, per capita consumption of poultry in Iran is approximately 25.16 kg (higher than the world average consumption).^28^

Pilgrimage and tourist attractions of the Mashhad city in Northeastern of Iran provides it with nearly 32 million pilgrims every year,^29^ and the amount of food which serves for this population, reveals the importance of foodborne illness in this city. 

In recent decades, many surveys have been conducted on the incidence of *C*. *perfringens* in raw and processed meat and poultry.^7 ^Sperner* et al. *reported that 20.00% of the fish and broiler meat samples as positive for *C. perfringens*.^30^ Higher incidence of *C*. *perfringens* has been reported by Miwa* et al.* in raw chickens meat as 84.00% and by Nowell* et al. *as 66.00%.^31^^,^^32^ Wen and McClane also reported the incidence of C. *perfringens* in 38.00% of raw chicken meats.^2^ In the present study, the total incidence of *C. perfringens* in broiler carcasses was 15.50%. The different results may be due to different meat processing methods or the method of sampling and bacterial isolation. In our study, the sampling method was rinsing the whole carcass, and the bacterial isolation method was enrichment in FTG medium followed by selective plating on sheep blood agar, and finally DNA extraction from suspected colonies and confirmation by PCR method.

Detection of *C. perfringens *toxin types and subtypes is critical for a better understanding of the epidemiology of *C. perfringens *infections and may be helpful in the development of effective preventive measures. The typing of *C. perfringens* strains was originally established based on neutralization of the pathological effect of each major toxin, both trypsin treated and untreated, with appropriate antisera in laboratory animal models.^33^ In diagnostic laboratories, this differentiation has been replaced by enzyme-linked immunosorbent assays (ELISA).^34^ Although ELISA allows reliable typing of *C. perfringens* isolates, the options for subtyping are limited. For example, so far no ELISA is available to detect the β_2_-toxin. In addition, high levels of enterotoxin have been shown only to be present during sporulation.^35^ As a consequence, sporulation of *C. perfringens* isolates has to be induced via specific cultivation methods to detect enterotoxin producing strains.^11^ These problems have been solved by genotyping of *C. perfringens* isolates. Various PCR protocols including multiplex PCR assays have been established to genotype *C. perfringens *isolates. Sensitivity and specificity are the two main characteristics of an efficient and practical technique existing in PCR. Rapidity is one of the major advantages of this method, so that bacterium identification and type determination lasts no longer than four hours. Hence, the toxicogenic strain in the sample can be identified by means of a rapid evaluation by PCR before it produces toxin. Genotyping of *C. perfringens *by PCR is a rapid and useful method and the use of a PCR variant, multiplex PCR, has enabled the simultaneous detection of the main toxins with the consequent saving of time.

Several studies reported that type A is the predominant type in poultry. The enterotoxins of type A have been reported to cause food-borne illness in humans.^2^^,^^36^ In contrary, our results showed that type C of *C. perfringens* was the most prevalent type in broiler meat samples. The same results has been reported by Poursoltani* et al.* which detected all 180 isolates of *C. perfringens* from wing, neck, liver and gizzard of broiler chickens in Mashhad, as type C by multiplex PCR.^37^

Only a small fraction (1.00 to 5.00%) of all *C. perfringens* isolates, mainly belonging to type A, carry the *cpe *gene.^38^ Intact *cpe *genes can also be found in some type C, D and E strains.^6^ Results of the present study showed that enterotoxigenic strains of *C. perfringens* were 25.00% which all of them belonged to type C isolates. Miwa *et al. *found that enterotoxigenic strains of *C. perfringens* were present in an average of 12.00% of poultry samples.^31^ Singh *et al. *reported the incidences of enterotoxigenic* C. perfringens *in 15.50% of poultry meat.^39^

Type C strains of *C*. *perfringens* are the only non type A strain that cause human disease,^40^ which is referred to as enteritis necroticans, also known as pigbel.^41^ Food poisoning by type C of *C*. *perfringens* is lethal in 25.00% of cases.^25^ The symptoms of *C. perfringens* type C food poisoning (necrotic enteritis) in human, start with abdominal pain and bloody diarrhea, and are followed rapidly by necrosis of the small intestine, caused mainly by the beta toxin, with contributions of additional toxins (*cpe* has been proposed as a possible contributor to the pathogenesis of human pigbel).^25^

In this study, all of the strains were identified as type A and C. The absence of types B, D and E is probably due to the origin of the samples which were broiler meats. In a study with samples of different origins, Songer and Meer reported 92.70% of isolate as type A; 0.10% of as type B; 4.50% of as type C; 2.10% as type D and 0.60% as type E.^15^ According to our results, it can be concluded that type C is the most predominant type in this region, and because this type causes more deadly illness in human, further investigations are required with larger sample sizes and more geographic distribution in Iran.
